# Practical Food of Real Value

**Published:** 1917-11-10

**Authors:** 


### PRACTICAL FOOD OF REAL VALUE.

### II. WHY AND WHERE HOSPITALS COST MORE.

By the House Governor of the London Hospital.

**[In our last issue, page 97, we gave the first portion of Mr. E. W. Morris's interesting notes of why and where hospitals cost more in war-time. The following is the conclusion of his practical and valuable remarks. We venture to hope that other hospital administrators, whose institutions may have followed a similar course, will send us a copy of any papers on the subject of hospitals' cost in war-time which they may have circulated.—Ed. "The Hospital."]**

### Fuel and Lighting.

I might point out that because coal is "up" everything that is made from coal is "up." Gas, for instance, and the scores of things made from coal tar (salicylate of soda, aspirin, carbolic acid). I note that "Fuel and Lighting" cost the hospital in the year 1913 (the year before the war) £8,522. Last year it cost us £11,770—an *increase* of over £3,000.

### Building Materials and Furniture.

In the surveyor's department the increases have been very marked. All that builders want is "up." And that is because these "building materials" are wanted for all sorts of things connected with the war; not only big things like steel girders, but little and less important things like screws and nails and wood planks, cement and sand. Common white-lead paint has jumped up from 40s. to 75s. a cwt. And some of the things cannot be had at any price. You would be surprised to hear how often the Cavell Home has been "held up." Once it was for steel girders; once for slates; once for sand for cement; once for wood (the wood had been bought and was in Gloucestershire, but we could not get the railway to bring it). And because timber is dear, everything made from timber is costly—furniture, for instance. We have just contracted for the furniture for the Cavell Home. It is to cost over £6,000. In peace-time it would have cost about £4,000.

Talking of furniture, the linoleum used in our wards has "gone up" to 6s. 2d. per square yard (from 4s. 6d.). Anything that is made from oil or fat is dear, because the Government wants all the oil it can get to make glycerine with, and from that—nitro-glycerine. So anxious is the Government to secure as much glycerine as possible that it is now impossible to buy glycerine for toilet purposes; every kind of fat or oil from which glycerine is made is precious. Linoleum is made from linseed oil (oleum lini, hence the name linoleum), and therefore we find it difficult to obtain linoleum. All fat, even the washings of plates, from every camp and hospital in France, is sent to Havre to be boiled up and purified, and is then sent to England—all to be used for extracting glycerine. It is because of the present value of fats of all kinds that soap has gone up from 18s. to 34s. 9d. per cwt. Fat is used for making soap.

### Increased Cost of Linen-Room Articles.

I have been having a talk with Miss Taylor, of the linen-room, on the cost of the articles for which she is responsible. Blankets have gone up from 6s. 11½d. each to 10s. 6d. each. That is because all manufacturers of blankets are hard at work making Army blankets, and have no time to bother about civilians. Sheets have gone up from 5s. to 12s. 6d., and, as a consequence, we have had to purchase cotton instead of linen sheets. Pillow-cases have gone up from 14s. 11½d. to 25s. per dozen; quilts from 5s. 9d. to 9s. 6d. each; flannel, for making flannel bandages, from 7½d. to 1s. 3d. a yard; bed mackintoshes were 4s. 6d. a yard, now 6s. 5d., pins used to cost 1s. 10d. per lb., now 3s. 11d. The cost of material for nurses' uniforms (three dresses) has advanced from 15s. 6d. to 24s. 2d. A summer cloak for the private nursing staff was 39s. 9d., now 69s. 6d.; a winter cloak has advanced from 42s. to 59s. 6d. The linen for nurses' caps used to cost 1s. 4½d. a yard, now it costs 2s. 11d. Tablecloths have gone up from 3s. 2d. per yard to 4s. 11d. a yard. Sewing cotton used to cost 32s. per gross of reels, now 59s. Hardly a thing in the linen-room but has advanced like this, even the small things like buttons and safety-pins and tape.

### Growth of Prices in Operation Theatres.

In the operating theatres one hears the same complaint. All goods made with rubber, such as tubing, catheters, and operation gloves, are advanced especially; the Government wants all the rubber it can buy. Glassware of all kinds costs more than double; that is because all our glass used to come from Germany; now our own manufacturers cannot supply what is wanted; bowls and jars and glass syringes, therefore, are all dearer, and so are photographic plates and *x*-ray tubes. All scientific apparatus is double the price, because we used to let the Germans make it for us, instead of learning to do it ourselves—microscopes, ophthalmoscopes, etc. Therefore breakages are of much more importance than in peace-time. And we must not think that because we are ready to pay for a thing when we break it there has been no waste. True, the *hospital* has been saved the expense of paying for the waste—but there has been waste. Something which is becoming increasingly difficult to obtain has been blotted out from future usefulness. It is lost. We must *not* break things at these times.

### The Tale of the Dispensary.

The dispensary has the same tale to tell. Aspirin used to cost 1s. 10½d. per lb., now 14s. 6d. per lb. Cod-liver oil was 2s. 10d. per gallon, now 15s. Olive oil was 4s. 8d. per gallon, now 11s. 9d. Paraffinum liquidum was 3s. 2d. per gallon, now 10s. 6d. Caffein was 13s. 3d. per lb., now 60s. Antipyrin was 6s. 5d. per lb., now 50s. Phenacetin was 2s. 7½d. per lb., now 60s. Bromide of potassium, which we use by the hundredweight, especially in the out-patient department for cases of epilepsy, was 1s. 6½d. per lb., it is now 6s. 9d. Saccharin was 22s. 6d. per lb., now £18 15s. per lb. Salicylate of soda was 1s. 5d. per lb., now 9s. Carbolic acid was 3⅜d. per lb., now 1s. 4¼d. We buy carbolic acid in tons at a time. A difference in its price of 1d. per lb. means that we have to give £9 more per ton. You see it has gone up more than twelve pennies per lb. Methylated spirit was 2s. 1d. per gallon, now 7s. 6d.

### The Trouble Due to our Careless Habits.

In nearly all these cases we deserve the trouble we are in. We let Germany make these things for us, and were content to buy them without knowing how they were

**PRACTICAL FOOD OF REAL VALUE—(*continued*).**

made commercially. Our inconvenience serves us—as a nation—right. The dressings used in the hospital also show a great increase in cost. Cotton-wool has advanced from 6d. to 1s. 7d. per lb.; grey wool from 4d. to 1s.; plain gauze from 5s. 3½d. to 9s. per 100 yards; boric lint from 9d. to 1s. 6d. per lb., and plain lint from 10d. to 1s. 7½d. **In the Laundry,** too, they are troubled with the prices always leaping up. The special soap they use has gone from £34 10s. to £56 per ton; starch from £32 10s. to £56 10s. per ton; borax from £22 to £36 per ton.

### The General Result and Outlook.

I have said enough to show how very serious the outlook is becoming, how criminal it is to allow any waste. The year before the war it cost £122,000 to "run" the "London" for a year. Last year it cost £155,000; an *increase* of £33,000. And that does not include anything spent on building. It is just "upkeep."

What are we to do? The public support the hospital splendidly, but they cannot support it, with all the other war charities claiming their help—they cannot support it to the amount of nearly £500 *a day*. We must at any rate do our bit, and that is to set our face against all waste. We must cut down hot water, gas, electric light, dressings, unnecessary drugs; we must guard against waste in food and coal, stamps and paper, soap and washing; in lotions, in spirit. The huge expenditure is made up of these little insignificant things. St. Paul's Cathedral is a collection of comparatively small stones, and little drops of water do make the mighty ocean. (By the by, this little hymn always irritates me. My nurse, a Hindoo lady, crooned it to me, a youthlet of six, and then ducked me in the sea. She had not told me the whole truth; the chilliness had not been referred to, nor the saltiness; and there were jelly-fish. It broke my faith in better things—at the early age of six).

### A Saving Crusade the Remedy.

Well, there are a thousand of us "employed" in hospital, and we will start on a saving crusade. I will agree that one person may waste as much as he or she likes, if you each will agree that that one person shall not be "you" or "me." So we will carry on. The work of the hospital must go on—the mothers and sisters and wives and sweethearts of the lads at the Front must be tended. If we do not do something to economise we shall have to close bit by bit of the hospital. We already *owe* £35,000, and we cannot go on borrowing indefinitely. Let us inconvenience ourselves a little more. *It is for England.*

